# Biomedical semantic text summarizer

**DOI:** 10.1186/s12859-024-05712-x

**Published:** 2024-04-16

**Authors:** Mahira Kirmani, Gagandeep Kour, Mudasir Mohd, Nasrullah Sheikh, Dawood Ashraf Khan, Zahid Maqbool, Mohsin Altaf Wani, Abid Hussain Wani

**Affiliations:** 1 University Institute of Computing, Chandigarh University, NH-05-Chandigarh-Ludhiana, Mohali, Punjab India; 2 Department of Computer Science, University of Kashmir, South Campus, Anantnag, Jammu and Kashmir India; 3 IBM Research, Almaden, 650 Harry Rd, San Jose, CA 95120 USA; 4 Thndr, The Office 3, One central, DWTC, Dubai, United Arab Emirates; 5 Department of Computer Science, Government Degree College Bemina, Srinagar, Jammu and Kashmir India

**Keywords:** Biomedical text summarizaion, Text summarization, Text semantics, Semantic models

## Abstract

**Background:**

Text summarization is a challenging problem in Natural Language Processing, which involves condensing the content of textual documents without losing their overall meaning and information content, In the domain of bio-medical research, summaries are critical for efficient data analysis and information retrieval. While several bio-medical text summarizers exist in the literature, they often miss out on an essential text aspect: text semantics.

**Results:**

This paper proposes a novel extractive summarizer that preserves text semantics by utilizing bio-semantic models. We evaluate our approach using ROUGE on a standard dataset and compare it with three state-of-the-art summarizers. Our results show that our approach outperforms existing summarizers.

**Conclusion:**

The usage of semantics can improve summarizer performance and lead to better summaries. Our summarizer has the potential to aid in efficient data analysis and information retrieval in the field of biomedical research.

## Introduction

The volume of biomedical documents has increased considerably since medical records were digitized. The patient-doctor interactions also lead to a considerable increase in biomedical papers. With more than 3500 documents added daily to different journals. Pubmed has around 36 million citations and abstracts of biomedical literature [1]. All these resources provide a valuable source of information to health practitioners, doctors, and researchers. However, retrieving information from this enormous knowledge base is cumbersome and time-consuming. Thus, text summarization is a potential solution to this information overload problem. Text summarization condenses this information for quick and efficient consumption.

Text summarization is the art of condensing lengthy textual documents into concise versions while retaining the original document’s core meaning and informational value. Doing so results in more digestible and easily comprehensible documents to the reader. This process facilitates the efficient handling of extensive documents. In formal terms, text summarization involves thoroughly analyzing and processing lengthy text documents to distill their essence for immediate consumption. This, in turn, enhances understanding and readability without sacrificing the document’s overall meaning and significance.

Text summarization is helpful as it serves the following purposes: Text summarization produces smaller documents, reducing the input documents’ size and hence a shorter reading time; text summarization helps produce reports used by commercial companies for easier decision-making; text summaries are useful in stock markets and reviewing financial statements; emails are easily comprehensible if email summarization is employed [2]; for e-learning systems, text summarization is highly beneficial; text summaries are highly useful in determining the polarity and sentiment of a document [3]. Hence, we find an excellent motivation to work on the text summarization and improve the process [4].

Text summarization is categorized into abstractive, extractive, and hybrid summaries depending on whether only a subset of sentences is chosen without any transformation of the selected sentences. If a subset of sentences is chosen that are representatives of the original document, without any modification of the original sentences, we call this extractive summarization. While as if some transformation is done for the selected subset of sentences, we call it abstractive summarization. We apply natural language generation tools to extractive summaries to obtain their abstractive version. Thus, the summarized sentences are distinct from the original set of sentences. The summarised document’s contents are different from the initial set of sentences in the original document. Both abstractive and extractive rules are applied in the hybrid summarization, hence the hybrid name for the summarization technique.

All three summarization techniques employ stylistic and syntactic rules to obtain summaries. Standard features used are the relative position of the sentence, length of the sentences, and presence of verb phrases and noun phrases. These features scale up well, but these methods miss out on an essential and characteristic feature of the textual structure, i.e., textual semantics. Semantics form an inherent and crucial feature of the input document but are overlooked by the existing summarizers in the literature. Hence, the existing summarizers are overlooking this aspect of the text. These summarizers assume that only statistical features are central to the summarization and thus miss out on semantics.

We use distributional semantic models to capture the semantics of the text. However, directly applying these models to biomedical text processing has limitations. These models are trained on general domain datasets; thus, their application on biomedical documents does not yield good results [5]. These documents include medical terminologies like abbreviations, synonyms, and hyponyms specific to the medical domain only. The word distributions of the biomedical documents differ from the other domain documents. Thus, applying the general domain semantic models to biomedical documents for text summarization achieves inaccurate results [6].

We introduce the application of *bio-semantic* models for text summarization. These bio-semantic models are extensions of distributional semantic models trained on biomedical datasets. In this study, we primarily focus on summarizing textual papers in the biomedical domain, which are an integral part of the vast biomedical literature.

In this paper, we hypothesize that the *bio-semantic* models that are extensions of the distributional semantic models result in better biomedical text summarizers. We implement text summarizers using these bio-semantic models. We use these models to capture semantics from the text by computing semantic coherence between two textual elements and then use them as a text summarization feature. Our evaluation of the results proves that the *bio-semantic* summarizer produces better quality summaries than the summarizers that do not use semantic models for the biomedical domain and achieve state-of-the-art results. The characteristic of our biomedical semantic summarizer is exciting and novel, considering that no summarization in the biomedical domain uses *bio-semantic* models for extracting semantic features and then using them for text summarization.

We propose an extractive summarization technique using *bio-semantics* models. The proposed *bio-semantic* summarizing system consists of four steps:

- Semantics of the text is used as a feature to obtain text summaries. We use *bio-semantic* models to obtain semantics. These models are used to obtain our novel *big-vectors*. Big-vectors are semantic bag-of-words extensions of the sentences. Each sentence of the input text document is fed to the bio-semantic model to obtain semantic transformation of the sentence. More precisely, the words of the sentence are given to the *bio-semantic* models to retrieve their word vectors. These word vectors for the given sentence are then concatenated to obtain a unique big-vector for the sentence.
- Next, we use a k-means clustering algorithm to cluster these big-vectors into different clusters.
- Next, ranking is performed on each cluster to obtain ranked sentences. We use our novel ranking algorithm that uses sentence scoring functions to obtain ranks for each sentence in the input document.
- The last step chooses the summary sentences among the highest-ranked sentences.

The term ’semantic bag-of-words’ refers to a representation that captures the semantic content of a document or sentence in a manner similar to the traditional bag-of-words model. In a standard bag-of-words approach, the emphasis is on word frequency, treating each word as an isolated unit without considering its semantic relationships with other words. In contrast, the ’semantic bag-of-words’ extends this concept by incorporating semantic information. Each ’big-vector,’ which is an extension of the traditional bag-of-words representation, not only considers the occurrence of individual words but also encodes their semantic meaning. This is achieved by leveraging distributional semantic models that analyze the contextual relationships between words. In essence, ’big-vectors’ serve as semantic extensions of traditional bag-of-words representations, providing a more nuanced understanding of the text by considering the semantic associations between words.

To validate our hypothesis, we performed experiments using two different *bio-semantic* models. We used the dataset used by Givchi et al. [5]. The dataset is publicly available.^1^

In the realm of biomedical text summarization, an extensive body of literature exists, with numerous studies delving into various aspects of this field. These investigations have yielded valuable insights into the challenges and opportunities surrounding the summarization of biomedical documents.

Within this context, it is imperative to recognize that while existing research has significantly contributed to the domain of text summarization, it also reveals some noteworthy gaps and unexplored avenues. Notably, many of the conventional summarization methods have primarily emphasized statistical and structural aspects of the text, often sidelining a pivotal facet–the intricate semantics of biomedical documents. Despite the rich and nuanced semantics intrinsic to the biomedical domain, previous summarization models have been limited in their ability to harness this semantic wealth.

As a result, a critical literature gap emerges that underscores the need for approaches capable of incorporating and leveraging the semantic intricacies inherent in biomedical texts. The vast and diverse terminology, including medical abbreviations, synonyms, and domain-specific word distributions, poses a unique challenge that necessitates the development of specialized methods. Existing summarizers, trained on more general datasets, often fall short when applied to the idiosyncrasies of biomedical documents.

These considerations lay the foundation for our study’s motivations. In this research, we aim to bridge the gap between conventional summarization methods and the distinctive semantic landscape of biomedical literature. By introducing *“bio-semantic”* models, extensions of distributional semantic models trained on biomedical datasets, we aspire to enhance the summarization process and elevate it to new levels of effectiveness. Our primary goal is to investigate the potential of these models in improving the quality of biomedical text summarization.

Furthermore, by conducting rigorous experiments and evaluations, we seek to provide empirical evidence that substantiates the superiority of bio-semantic summarization over conventional approaches. Our pursuit of state-of-the-art results is driven by the compelling motivation to offer the biomedical community an advanced summarization tool that optimally captures and conveys the intricate semantics of biomedical documents.

By addressing these literature gaps and motivations, our study contributes to the ongoing evolution of text summarization in the biomedical domain and presents a novel approach that is tailor-made for the unique characteristics of this field.

The results of our summarizer are evaluated using *ROUGE* (Recall-Oriented Understudy for Gisting Evaluation). ROUGE produces results using the comparison of ground truth (human summaries) and system-generated summaries. It quantifies results by generating different metrics like precision, recall, and f-score. Three different ROUGE scores namely ROUGE-1, ROUGE-2 and ROUGE-L are reported for evaluation of our results. We have used these specific ROUGE types since they are standard metrics for evaluating text summarization models, and many previous studies have reported results using these metrics. Their widespread adoption enables easy comparisons with existing literature and benchmarking against other models.

We also compare our system with other biomedical summarizers found in the literature. The results confirmed our hypothesis and effectively described the competitive effectiveness of our proposed system.

The rest of the paper is described in the following manner. Section “Related works" discusses exhaustive state-of-art text summarization, Sect. “Methodology” discusses the methodology employed, Sect. “Experimental setup and results”  discusses the results and comparative analysis, and we finally conclude in Sect. “Conclusion and future work”.

## Related works

This section covers various aspects of text summarization, including the techniques employed by automatic summarization systems, their limitations, and the benefits they offer compared to others. A comprehensive examination of various methods, techniques, and feature selection is conducted to draw conclusions and identify remaining challenges.

### Unsupervised techniques

The unsupervised approach of text summarization uses statistical features of text for summarizing the documents. It is the most used approach for summarization. The earliest attempt to obtain text summaries was made by Luhn in 1950. Luhn worked with the assumption that a document consists of various concepts and terms. The most important terms for summarization are the most frequent terms used in the document. This leads to the conclusion that the more frequently the term is in the document, the higher its importance for the summary. Luhn also concluded that these frequent terms are thematic terms or descriptive terms. Luhn thus proposed that such terms should be included in the summaries [7].

Edmundson et al. [8] extended Luhn’s work by incorporating extra features that improve the score of relevant sentences for text summarization. They selected the following characteristics to score the sentences in the documents: (1) word frequency; (2) the number of title word occurrences in a sentence; (3) Position of the sentence in the document; the more important the sentence, the higher it is in the document. Baxendale et al. [9] laid the framework for abstract summarizers. The paper mentions that summaries can be created by adding sentences from those not in the document’s text.

The unsupervised technique employed in the literature includes the works like [10–12]. They use different features to rank sentences and produce extractive summaries. These features are statistical and used to rank sentences for summarization. Mihalcea et al. introduced *textrank* [13], an algorithm that uses several statistical features and one specialized function that calculates a weighted sum for calculating the similarity between sentences [14].

### Query based text summarization

In this technique, the summaries are generated by a scoring mechanism. The sentences that contain the query terms are scored higher and thus obtain higher ranks. These high-ranked sentences constitute the summary of the input documents. More specifically, this approach centres around the user query terms and their extensions. The user-input query terms form the basis of this type of summarizing model.

Higher-scoring sentences and other structural elements are the summarizer’s output. With query-based summarization, the output summaries may have extracts composed of different portions or sections of the input text; hence, summaries are the union of these other extracts. In their paper [15] uses long text queries to solve word sense disambiguation problems in Urdu information retrieval system.

To overcome the issue caused by the incomplete information within the initial queries, the paper [16] proposed to combine a query expansion method with a graph-based summarization strategy. The input text document used as an input for summarization is used for query expansion rather than any external sources like WordNet. The paper [17] uses sentence-to-sentence and word-to-sentence mapping for query expansion. They evaluated their system using DUC 2006 and DUC 2007 datasets. The evaluation of the results confirms that their system performs better than the system that does not use query expansion. The Support Vector Regression (SVR) is used by [18] to find important sentences in the input document for summary generation in response to the user query. [19] designed a query-based text summarizer using word sense disambiguation. They expand the queries using common sense knowledge. [20] uses query-based summarization to obtain slide-based presentations for the research articles.

### Machine learning based approaches

Recent advances in machine learning algorithms have made automatic text summarizing (ATS) applications possible. The techniques employed by these algorithms include identifying and extracting suitable feature and their corresponding application for the design of ATS. These algorithms create a model and then use that for the ATS. The learned model then determines the sentences that will be part of the summaries. There are various ATSs defined in the literature using machine learning. Several studies have utilized different methods to generate extractive summaries. For instance, [21] employed the Support Vector Machines (SVM) and the Naive Bayes model with relevant, topic, event, and content features. Meanwhile, [22] developed an extractive summarizer using machine learning techniques and statistical and linguistic data directly from the source textual document. [23] utilized hidden Markov chains (HMMs), and [24] enhanced extractive summary outcomes by ordering sentences in the documents.

### Neural networks based approaches

Deep learning has gained popularity due to recent technological advancements and decreased memory costs. When appropriate training data is available, neural network summarizers perform better than traditional automatic summarizers with minimal human intervention. In their paper, [25] presents an overview of all the neural network algorithms that are used as state-of-art for summarising text. Researchers have used neural networks in various forms to develop text summarization systems. For example, [26] utilized continuous vectors based on neural networks to create an extractive summarizer and achieved better results. The first abstractive summarizer using CNNs was introduced by [27]. [28] built on this work by creating an abstractive summarizer using CNNs and other neural networks. An RNN with an attentional encoder-decoder was used by [29] to generate an abstractive summary. COPYNET, a sequence-to-sequence learning technique that copies text segments at specific intervals, was introduced by [30]. The off-vocabulary problem was tackled by [31] using a neural network and a pointer-generator approach. The Chinese corpus was used by [32] to generate a summary. [33] described a neural network that uses a distraction strategy to allow users to focus on different input parts. A semantic relevance-based neural network was used by [34] to create semantically important summaries of Chinese data. Finally, [35] used a bi-directional LSTM encoder to generate an extracted summary.

### Graph based approaches

[36] Graph-based techniques used supervised and unsupervised learning schemes to create extractive single-document summaries. The goal is to find relevant sentences by extracting statistical aspects from these two approaches and then use graphs to determine sentence coherence. [37] computed word similarity adjacency networks to arbitrate authorship. They used text as a graph to identify the author. [38] employs multi-layer graph approaches for summarising several documents, where nodes represent sentences and edges represent the coherence between the two sentences. In their paper [39] achieved good results on multi-lingual datasets.

### Biomedical summarization

With the increased focus on domain-specific NLP applications, automatic text summarization for biomedical documents has recently gained much attention. Various biomedical text summarizers exist in the literature that achieves good results. [40] designed a deep bidirectional language model that uses contextual embeddings. The system works on the context and achieves better results than the baselines. [41] uses reinforcement learning-based biomedical summarization to summarize biomedical papers from their abstracts as headlines. These headlines are the domain-aware abstractive summaries of the input papers. [42] uses BERT and openAI GPT-2 to design the biomedical text summarizer. The designed system uses keywords extracted from the original text articles. [43] uses transfer learning employing text-to-text summarization set up. The model uses RNN with an attention mechanism to perform abstractive summarization of the medical documents.

These state-of-the-art systems achieve efficient results and contribute to the knowledge base, leading to efficient text summaries. However, all these systems have some drawbacks. Neural network-based summarizers produce good summaries; however, they require large volumes of data and enormous computation time. Machine learning-based systems require feature identification and selection for performing competitively. Unsupervised and graph-based methods tend to miss out on essential and fundamental features, i.e., semantics and meaning of data. Thus, we propose a domain-specific text summarizer for the biomedical domain that captures the text’s underlying meaning and semantics. The proposed approach uses neural networks and unsupervised algorithms to summarize biomedical documents. The evaluation and analysis of our results confirm that using semantics as a feature for text summarization improves the system’s performance as a whole.

## Methodology

**Fig. 1:**
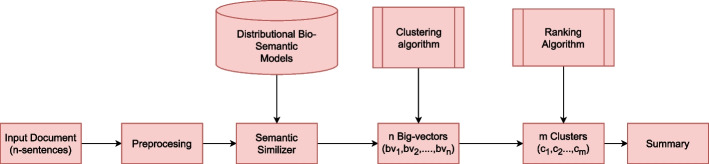
Overall working of the system

In this section, we describe our methodology for constructing our ATS for biomedical documents using *bio-semantic* models. The novelty of our approach is using bio-semantic models for utilizing semantics as a feature for ATS. We employ bio-semantic models for extracting semantic features and then use these features along with other stylistic and statistical features. Figure 1 shows the architecture of our proposed approach.

More specifically, our proposed approach consists of the following steps: (1) Preprocessing text for text normalization and inconsistency removal; (2) Distributional semantic models used to capture text’s semantics;(3) The vectors generated by distributional semantic models are combined through our novel vector generation algorithm known as the big-vector generation algorithm to produce dense semantic extensions of the input sentences.; (4) We then cluster the semantically similar sentences together in a single cluster using the clustering algorithms. The single cluster is a coherent representation of the most similar sentences.; (5) Ranking algorithm to obtain scores for each sentence from each cluster; and (6) Normalization of scores for efficient sentence extraction to obtain the summary.

### Preprocessing

Preprocessing is an essential step in our system. The purpose of preprocessing is to clean the data and remove inconsistencies. Preprocessing is performed to make data uniform and processing-ready. We perform the following functions during preprocessing:

- **Remove unnecessary parts from papers:** The unnecessary elements from the paper, like abstract, title, tables, figures, and references, are unnecessary regarding summarization. Thus, they are removed from input documents before our system processes them.
- **Tokenization:** The input text is processed in the same units for uniformity. We break text into words for efficient processing.
- **Removing punctuations and numbers:** Numbers and punctuations convey no meaningful information content and are thus removed from the input text.
- **Lowercase:** All the text is converted to lowercase for efficient processing.
- **Lemmatization:** Lemmatization is a text normalization technique used in Natural Language Processing (NLP). It’s used to identify word variations and determine the root of a word. Lemmatization groups different inflected forms of words into the root form, which have the same meaning. For example, a lemmatization algorithm would reduce the word “better” to its root word, or “lemma” or “good”. The words extracted during tokenization are then transformed into their base or root form through lemmatization, ensuring consistency and aiding in subsequent analysis. This step is facilitated using the Stanford Core NLP package [44].

### Capturing semantics using distributional bio-semantic models

*Bio-semantic* models are the extensions of distributional semantic models. These models are domain-specific and limited to the biomedical domain. These models work on the principle of distributional hypothesis. According to this hypothesis, words used in the same context tend to have similar meanings. These models do not require any lexical and linguistic analysis. Furthermore, these models are independent of external information to obtain semantics.

*Bio-semantic* models are the pre-trained language representational models for the bio-medical domain. They are trained in the biomedical domain corpora like PubMed abstracts and PMC full-text articles. These models are used in various biomedical NLP applications like Named Entity Recognition, Question-Answering systems [45, 46], Neural Machine Translation [47], and Relation Extraction [48].

Our approach uses word embeddings to build semantic models that capture the coherence between two textual elements. These models create word embeddings by using statistical computations on the context in which the word appears, considering the words that occur close to the target word. The resulting dense vector representation of the target word is called word embedding. The coherence between two elements is measured using these word embeddings. Using these models in text summarization helps capture the meaning and relationships between sentences, leading to a summary that preserves the semantic coherence of the original text. Word embeddings are high-dimensional real-valued vectors generated for each word. They have been found to be helpful in NLP tasks such as text classification, sentiment analysis [49], and machine translation. In high-dimensional vector spaces, these representations and their geometric properties can help determine the coherence of various word usages. This results in the observation that words close in high-dimensional vector spaces are syntactically and semantically similar.

We have employed two distributional bio-semantic models, namely BioBERT [45] and a biomedical extension of Word2Vec [50], in various experiments to validate our hypothesis. These models are extensions of distributional semantic models. Word2Vec, a two-layer neural network model, excels at producing high-quality text semantics. It accomplishes this by transforming words into high-dimensional vector space embeddings, with the model taking a word as input and generating a semantic-rich vector as output–a name that aptly describes its functionality.

Word2Vec offers two architectures: Continuous Bag of Vectors (CBOW) and Skip-gram. The CBOW model predicts a word from its context, while Skip-gram is used to predict the context surrounding a given word. Moen et al. [50] crafted 200-dimensional vectors using Word2Vec [51], leveraging all publication abstracts from PubMed and full-text documents from the PubMed Central Open Access subset. They employed a skip-gram model with a window size of 5, hierarchical softmax training, and a frequent word subsampling threshold of 0.001 to construct these 200-dimensional vectors.

BioBERT [45] (Bidirectional Encoder Representations from Transformers for Biomedical Text Mining) represents a specialized extension of BERT [52] tailored for the biomedical domain. In the subsequent subsection, we introduce our novel Big-vector generation algorithm, which plays a crucial role in capturing semantic information from the input biomedical documents.

#### Big-vector generation

Our big-vector generation process introduces a novel algorithm for constructing big-vectors from the vectors generated by distributional bio-semantic models. To achieve this, we feed all the words in the input text into the bio-semantic models. This allows us to obtain concatenated vectors for each sentence, effectively creating a comprehensive bag of words represented by a single vector.

Let’s denote $\beta (w)$ as a function responsible for retrieving a list of the top ’m’ similar words from a given bio-semantic model. Mathematically, this function can be expressed as $w' = \beta (w) = w'_1 \oplus w'_2 \oplus \ldots \oplus w'_m$. For a sentence comprising a sequence of ’k’ tokenized words represented as $W = \{w_1, w_2, w_3, \ldots , w_k\}$, where ’w’ represents a word from the sentence. We construct a big-vector *BGV* by concatenating the respective top ’m’ similar words for each word. In other words, *BGV* can be defined as $BGV = \{\beta (w_1) \oplus \beta (w_2) \oplus \ldots \oplus \beta (w_k)\}$.

This process allows us to create meaningful big-vectors that capture the essence of the input text and serve as valuable semantic representations for our summarization tasks.

This rich semantic vector is then fed to the clustering algorithm to obtain different clusters representing all the semantic information for that cluster [53].

### Clustering

In this phase, we employ the K-means clustering algorithm to group semantically rich big-vectors obtained from the input sentences. These big-vectors represent an extension of the sentences as a semantic bag of words. It’s essential to note that these vectors result from applying distributional semantic models to capture the intricate semantic structures within the text.

The K-means clustering algorithm [54] is utilized to organize the big-vectors into distinct clusters. However, it’s crucial to highlight that the choice of clustering algorithm and parameters plays a pivotal role in shaping the summarization outcome. The algorithm aims to divide the sample space of big-vectors into semantically meaningful clusters, ensuring that each cluster comprises sentences with similar semantic content.

While the semantic content is a priority during clustering, the specific similarity metric employed is an important consideration. In this context, we focus on capturing the semantic relationships between sentences without explicitly considering word positional information.

The clustering process serves two key purposes in enhancing the summarization process. Firstly, it promotes diversity in the summary by ensuring the representation of various semantic dimensions in the original document. Secondly, it facilitates the efficient extraction of the most salient sentences from each cluster, contributing to the overall coherence and informativeness of the final summary.

**Algorithm 1:**
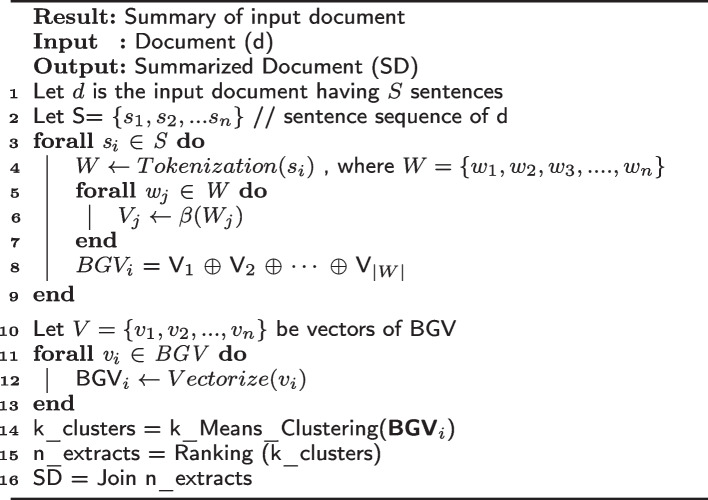
Summarizing algorithm

### Ranking algorithm

Our novel ranking algorithm aims to assign sentence ranks according to different surface-level features. The features that we include in our system are the following:

- Sentence length: The length of the sentence is directly proportional to its importance. We use sentence length as a feature for our summarizer.
- Sentence Position: the position of the sentence describes its importance. The more important the sentence, the higher its position. Thus, using it as a feature for ranking is important for summary generation. The sentence position score is calculated as follows:
  $$\begin{aligned}s^{p}_{i}=1-\frac{s_{i} -1}{|S|}\end{aligned}$$
  where, $s^{p}_{i}$ is the sentence position score of $i\text{th}$ sentence of the input document *S*, and |*S*| is the number of sentences in the input document.
- Frequency (TF-IDF): TF-IDF is a crucial distinguishing feature in any text summarization system. It locates the most important terms in any document. The ranking algorithm uses this attribute to find the essential words and, thus, sentences in the textual content. The ranking algorithm calculates the TF-IDF score of individual words and then uses the score of individual words to calculate the TF-IDF score of sentences. TF-IDF of the sentence is the sum of the TF-IDF scores of the individual words in the sentence. Formally, $TF-IDF$ of a sentence $s_i$ is calculated as:
  $$\begin{aligned} s^{tf}_{i} = \sum _{w \in s_i} t_f(w)\end{aligned}$$
  where the $t_f(w)$ is a function which gives the $TF-IDF$ score of a word *w*.
- Verb phrase noun phrase: The most important sentence in the input text has both a noun and a verb phrase. An imperative sentence contains one of these two phrases, and the precedence of either is ranked higher by our ranking algorithm.
- Proper nouns: Proper nouns contain direct referrals to the subject. Hence, their existence in a sentence increases the importance of the sentence.
- The cosine similarity measure determines the similarity between two documents. In this algorithm, cosine similarity is a feature used in the ranking process. The cosine similarity between two sentences is calculated, and the higher the cosine similarity, the higher their rank. The average cosine similarity of the $i^{th}$ sentence, $s^{c}{i}$, is calculated by taking the sum of cosine similarity between sentence *i* and all other sentences, divided by the total number of sentences in the document (|*S*|). The equation is as follows: $s^{c}{i} = \frac{ \sum _{j=1, j \ne i}^{|S|}c(s_i,s_j)}{|S|}$, where $c(s_i,s_j)$ is the cosine similarity between sentence *i* and *j*.

The total score of the sentence is then calculated by summing the individual normalized scores of each sentence. Algorithm  1 explains the proposed system algorithmically. Table 1 shows an example of scores calculated for some sentences using the ranking algorithm.

**Table 1:** Scores of ranking algorithm on example sentences

#	Sentences	Feature scores					
		TF	TW	C	SL	PN	T
1	The drug reactions for Alkaline phosphate and serum amylase are usually rare and do not affect normal and healthy adults.	0.425	0.80	0.162	0.048	0.032	0.812
2	Experiments conducted in this study were controlled by introducing adults aged 35-40.	0.149	0.87	0.231	0.032	0.043	0.421
3	Assessments of different drug reactions were considered by including various parameters obtained using different controlled settings.	0.462	0.42	0.252	0.051	0.01	0.623
4	Drug and their antidotes are analyzed using different lab slides prepared by using microwaves	0.231	0.12	0.104	0.104	0.012	0.423

*TF* Term frequency-inverse document frequency, *TW* Token weight, *C* Cosine similarity, *SL* Sentence length, *PN* Proper nouns, *T* Total score

Connecting words are used to establish a relationship between two sentences. Thus, these words connect two consecutive sentences. The words like however, moreover, but, and because are examples of connecting words. The morphology of a sentence with these connecting words is such that the meaning of two sentences is incomplete without its connecting sentence. Thus, if a sentence starts with a connecting word chosen by our algorithm, we make including a connecting sentence essential for the summary, irrespective of its rank. After the execution of the ranking algorithm, we have sentences sorted in some order according to their ranks. The system then selects the best-ranked sentences for inclusion in the summary.

**Fig. 2:**
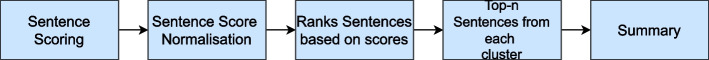
Functioning of ranking algorithm

This ATS (Automatic Text Summarization) algorithm aims to reduce redundancy in summaries by identifying and eliminating semantically similar sentences. It does this by clustering semantically similar sentences into a single cluster. If two sentences in the same cluster have almost similar ranking scores, it implies that they convey similar meanings and are included only once. The sentence with a higher sentence position score will be selected in summary. This feature is important in producing summaries of long technical papers where authors repeat sentences differently. Still, despite their high ranks, the algorithm can identify and discard them from the summaries.Figure 2 shows the overall working of the ranking algorithm.

## Experimental setup and results

This section describes the datasets and the experiments to evaluate the proposed algorithm and presents the recorded results.

### Dataset

To the best of our knowledge, no proper dataset consisting of articles and their corresponding human-generated summary, also known as reference summary, exists for the biomedical text-domain. Several types of research exist in literature where authors have used biomedical papers from various sources like PubMed and BioMed central and their corresponding abstracts as reference summaries [55, 56]. We have also used a similar kind of approach in this paper. The dataset we use is curated by [5]. In this dataset, 400 random articles are downloaded from BioMed Central. The paper’s text is used as input files, and their corresponding abstracts act as reference files for comparison with the system-generated summaries. The dataset is publicly available for download.^2^ The reason for using this as our dataset is that the experiments can be easily replicated. Table 2 shows the statistical information of the dataset used in this paper.

**Table 2:** Statistical information of the Dataset used

No. of sentences per document			No. of words per document		
Min.	Max.	Avg.	Min.	Max.	Avg.
50	610	163	836	10128	5511

### Baselines

We evaluate our approach against the following sate-of-art baselines

- **Graph-based abstractive biomedical text summarization** [5] Givchi et al. [5] use the graph-based technique to generate extractive summaries of biomedical text documents. They use the concept of frequent itemset mining to identify frequent concept sets that are then represented as graphs, and the shortest path is used to obtain extractive summaries.
- **Genism** [57] The Gensim summarizer is based on the **TextRank** algorithm [13], which is a graph-based ranking algorithm used for summarization. In **TextRank**, the importance of a sentence is determined recursively based on the global state of the graph. The algorithm works by voting, where a vertex that is linked to other vertices receives votes, and the more votes it receives, the higher its rank.
- **PyTextRank** is a Python implementation of the **TextRank** algorithm for graph-based summarization, just like Genism, and it produces text summaries using feature vectors. The main difference between the two is that PyTextRank uses spaCy for natural language processing and graph building, while Genism uses its own implementation.
- **PKUSUMSUM** [58] is a versatile summarization platform written in Java, offering support for multiple languages and incorporating ten different summarization methods. It caters to three primary summarization tasks: Single-document summarization, Multi-document summarization, and Topic-based Multi-document summarization. The platform features a diverse and stable set of summarization techniques, making it a reliable reference system for our evaluation. Noteworthy summarization methods included within the platform are **Centroid**, **LexPageRank**, and **TextRank**. For our evaluation, we have employed the single-document summarization approach, leveraging the **LexPageRank** method for summarization.

The baseline systems used in this paper are all state-of-art ATS. These systems achieve good results and are used for comparison with several other systems in the literature. These systems employ different kinds of summarization techniques, and thus, we can perform an exhaustive and comprehensive comparative analysis of our system. Furthermore, all these systems are publicly available; thus, experiments described in our paper are easily repeatable.

### Summary evaluation

Out of the 400 input documents in the dataset, we randomly chose 30 input documents to evaluate our system and compare the results with the baselines. We generate summaries for all the 30 papers selected using our proposed approach and the baselines. For extensive evaluation, short and lengthy summaries are generated by fixing the length of summaries to 15% and 25% of the original input document, respectively.

For evaluation, we use *ROUGE* (Recall-Oriented Understudy for Gisting Evaluation) automatic summarization evaluation toolkit [59]. ROUGE is publicly available and can be downloaded.^3^ It consists of a set of parameters for assessing automated summaries of texts. ROUGE generates four distinct types of ROUGE metrics, namely ROUGE-1, ROUGE-2, ROUGE-L, and ROUGE-SU4 values, by comparing summaries at various levels of granularity. ROUGE-1(2) uses unigrams(bigrams) for measuring coherence between the system summaries and the reference summaries; ROUGE-L uses the summary level Longest Common Sub-sequence (LCS) to match the coherence between the reference and system-generated summaries, and ROUGE-SU4 uses both skip-grams and unigrams for the measurements. ROUGE evaluates summaries of our system and the baselines by comparing them against the base truth, i.e., reference summaries, and computes different matrices. These matrices are *Precision*(*Pr*), *Recall*(*Rc*) and $F-score (Fs)$. The results for all 30 input documents are averaged and presented in the Tables 3 and 4. Table 3 shows the results for 15% summary length, and Table 4 shows the results for 25% summary length.

**Table 3:** Averaged summarization results of 15% summary length

Metric	Rouge Type	Bio-BERT	Bio-Word2Vec	Gensim	Graph-Based	PyTeaser	PKUSUMSUM
${\textbf {Pr}}$	ROUGE-1	** 0.65** (0.09)	0.58 (0.10)	0.29 (0.07)	0.52 (0.07)	0.41 (0.07)	0.24 (0.05)
	ROUGE-2	** 0.39** (0.06)	0.32 (0.05)	0.13 (0.04)	0.26 (0.05)	0.16 (0.11)	0.23 (0.14)
	ROUGE-L	**0.94** (0.09)	0.87 (0.10)	0.40 (0.07)	0.68 (0.06)	0.44 (0.024)	0.32 (0.28)
${\textbf {Rc}}$	ROUGE-1	0.26 (0.10)	** 0.26** (0.09)	0.18 (0.06)	0.23 (0.06)	0.15 (0.07)	0.18 (0.04)
	ROUGE-2	**0.16** (0.08)	0.13 (0.03)	0.16 (0.03)	0.11 (0.03)	0.19 (0.01)	0.09 (0.11)
	ROUGE-L	**0.31** (0.09)	0.28 (0.05)	0.17 (0.03)	0.26 (0.05)	0.19 (0.03)	0.23 (0.04)
${\textbf {Fs}}$	ROUGE-1	**0.36** (0.10)	0.35 (0.05)	0.20 (0.06)	0.31 (0.05)	0.20 (0.06)	0.22 (0.03)
	ROUGE-2	**0.22** (0.08)	0.18 (0.05)	0.14 (0.03)	0.15 (0.04)	0.18 (0.04)	0.17 (0.03)
	ROUGE-L	**0.46** (0.10)	0.42 (0.06)	0.24 (0.03)	0.39 (0.06)	0.21 (0.04)	0.28 (0.34)

Bold value represents the highest value for that observation

**Table 4:** Averaged summarization results of 25% summary length

Metric	Rouge type	Bio-BERT	Bio-Word2Vec	Gensim	Graph-based	PyTeaser	PKUSUMSUM
${\textbf {Pr}}$	ROUGE-1	** 0.88** (0.12)	0.81 (0.11)	0.52 (0.11)	0.75 (0.11)	0.64 (0.11)	0.65 (0.23)
	ROUGE-2	** 0.53** (0.08)	0.46 (0.08)	0.17 (0.08)	0.41 (0.08)	0.29 (0.08)	0.35 (0.18)
	ROUGE-L	**0.79** (0.07)	0.71 (0.08)	0.41 (0.08)	0.64 (0.08)	0.53 (0.08)	0.68 (0.23)
${\textbf {Rc}}$	ROUGE-1	0.30 (0.14)	0.31 (0.13)	0.29 (0.13)	0.32 (0.13)	**0.36** (0.13)	0.28 (0.15)
	ROUGE-2	0.16 (0.08)	0.17 (0.08)	0.15 (0.05)	0.17 (0.05)	** 0.21** (0.05)	0.17 (0.13)
	ROUGE-L	0.27 (0.08)	0.28 (0.05)	0.26 (0.05)	0.28 (0.05)	** 0.32** (0.05)	0.18 (0.07)
${\textbf {Fs}}$	ROUGE-1	**0.43** (0.10)	0.43 (0.08)	0.34 (0.06)	0.41 (0.13)	0.39 (0.10)	0.28 (0.15)
	ROUGE-2	**0.32** (0.10)	0.32 (0.05)	0.15 (0.07)	0.24 (0.05)	0.23 (0.04)	0.19 (0.15)
	ROUGE-L	**0.46** (0.08)	0.42 (0.06)	0.32 (0.05)	0.35 (0.06)	0.40 (0.04)	0.26 (0.23)

Bold value represents the highest value for that observation

The evaluation of the results obtained using bio-semantic models for capturing semantics for the text summarization process proves that the bio-semantic models aid in producing better summarization results. We have used evaluation techniques similar to the one employed in [60] and [61]. The results show that the proposed model performs better than the baselines. The baseline models do not employ semantic features, so our results are better than the baselines. Our system achieves better results in terms of precision and F-scores using both short and long text summaries. These results confirm our hypothesis that using bio-semantic models to capture the semantics of bio-medical documents and using these semantic features for text summarization improves the performance of bio-medical text summarization.

**Fig. 3:**
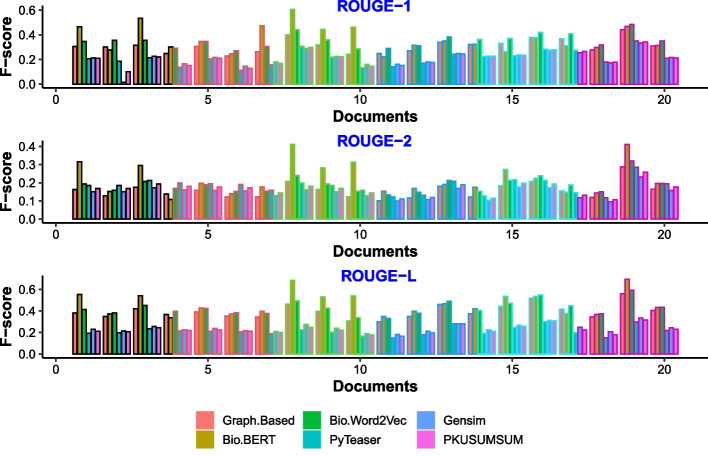
F-Score for 15% summary length

**Fig. 4:**
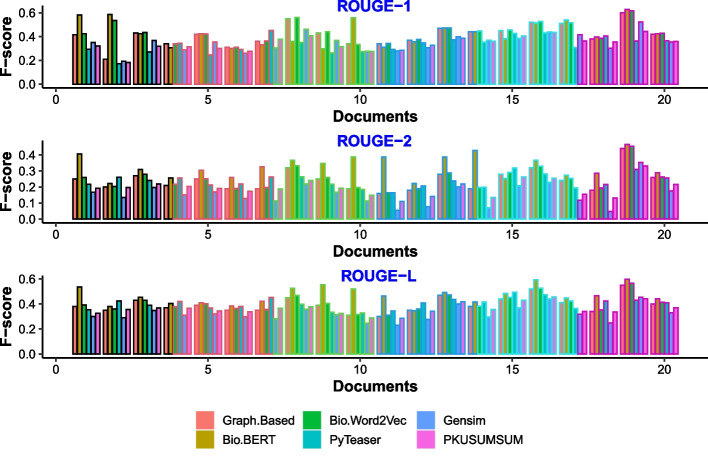
F-Score for 25% summary length

We obtained both long and short text summaries for extensive evaluation. The length of the summaries for the longer version is restricted to 25%, and for more concise summaries, we limited the length to 15% of the original text length. We obtained the macro-average of the precision for 25% summary length; we got 88%, 53%, and 79% and 81%, 46% and 71% for Rouge-1, Rouge-2, and Rouge-L using Bio-BERT and Bio-Word2Vec respectively.

The F-score for Rouge-1, Rouge-2, and Rouge-L obtained for 25% summary length are 43%, 32% and 46% using Bio-BERT and 43%, 32% and 42% using Bio-Word2Vec. These scores are higher compared to the baselines. These scores are statistically significant. We measured the statistical significance using a p-test. The baselines employ different summarization techniques and algorithms and are currently state-of-the-art in text summarization. Thus, comparing them leads us to exhaustively and comprehensively evaluate our system. The evaluation and comparison with the baselines prove the competitive efficacy of our proposed approach. Also, the baselines are open source, and thus, experiments are easily repeatable.

Furthermore, the evaluation and comparison of results obtained using 15% summary length show our system achieves consistently good performance on shorter length summaries. Our precision, recall, and f-score are better than the rest of the baselines.

The competitive superiority of our system is attributed to using semantic features for the text summarization process. Hence, we conclude that using semantics as a feature for summarization improves the system’s performance. The proposed system using the *bio-semantic* model outperforms the baselines with better f-scores and precision. The lower recall value in some cases is attributed to the fact that our system discards some statically significant sentences that are semantically similar to some sentences already chosen.

Figures 3 and 4 show our system’s comparison with the baselines on 30 randomly selected papers. Figures show the f-scores of these 30 documents obtained for different rouge matrices.

### Statistical testing

In addition to presenting our experimental results, we conducted a statistical paired t-test to establish the significance of the outcomes produced by our proposed model. To do this, we started by assuming the opposite scenario, known as the null hypothesis. According to the null hypothesis, our proposed method did not yield statistically significant results when compared to the baseline methods. We carried out the paired t-test on a set of 30 summary samples where the results of our proposed model were compared with those of the baseline algorithms. The results of this statistical analysis clearly indicate that our proposed model consistently selected more semantically-rich sentences compared to the baseline models.

To further ascertain whether the results obtained from our proposed algorithm are statistically meaningful or merely coincidental in comparison to the baseline methods, we conducted a significance test. During this test, we set a significance level of 5%. In simple terms, we ran 30 samples with a 95% confidence level. The initial null hypothesis, which suggested no significant difference between our proposed model and the baseline model, was rejected in favor of an alternative hypothesis. This alternative hypothesis implies that the outcomes produced by our proposed model are indeed significantly different from those of the baseline model. Therefore, our model consistently generates effective results.

Our analysis of F-score values for ROUGE-1, ROUGE-2, and ROUGE-L metrics reveals that the compression rates of 0.05% to 0.5% considered in our experiments contribute to the creation of coherent, less redundant, and diverse summaries, with a strong emphasis on semantic attributes. The *p*-value, as illustrated in Table 5, represents the proportion of observations where our model outperformed the baselines.

**Table 5:** *P*-value under *T*-test

Metric	Rouge-1	Rouge-2	Rouge-3
Bio-BERT vs. Gensim	0.0069	0.0054	0.0085
Bio-Word2Vec vs. Gensim	0.0074	0.0083	0.0060
Bio-BERT vs. Graph-Based	0.0078	0.0063	0.0074
Bio-Word2Vec vs. Graph-Based	0.0082	0.0086	0.0064
Bio-BERT vs. PyTeaser	0.0069	0.0061	0.0085
Bio-Word2Vec vs. PyTeaser	0.0082	0.0065	0.0074
Bio-BERT vs. PKUSUMSUM	0.0069	0.087	0.0073
Bio-Word2Vec vs. PKUSUMSUM	0.0056	0.0062	0.0085

## Conclusion and future work

As stated in the introduction, the rationale behind this research is to use bio-semantic features for the text summarization process to improve the quality of summaries.

This paper presents a novel way of extracting and using these bio-semantic features for text summarization. We use bio-semantic models to capture these features and then use these features to produce high-quality summaries for the bio-medical documents. According to evaluation and comparative analysis, our summarizer’s appropriateness, reliability, and scalability show that our proposed approach performs better than the baselines. The main conclusions of our paper are: (1) Usage of bio-semantic models to capture semantics in the bio-medical domain is an excellent choice for capturing semantics. (2) The semantic features work well in improving the efficacy of the text summarization process. (3) The usage of semantic features improves the text summarising algorithms’ overall accuracy. The primary disadvantage of these models is that they are computationally expensive.

Our future research will deal with (1) Improvement of ranking algorithms by exploring more semantic features to be incorporated into our ranking algorithms, as semantic features tend to improve overall system performance; (2) testing the technique on more than one dataset; (3) Using BLEU (bilingual evaluation understudy) for evaluation along with the ROUGE.

## Data Availability

The dataset used and analyzed during this study is included in the paper [5] and is publicly available and can be downloaded from https://github.com/azadehgivchi/abs_biomed_summary (last accessed on April 19, 2023).

## Footnotes

1 https://github.com/azadehgivchi/abs_biomed_summary/tree/main.

2 https://github.com/azadehgivchi/abs_biomed_summary/tree/main.

3 https://github.com/kavgan/ROUGE-2.0.

## References

[1] Moradi M, Ghadiri N (2018). Different approaches for identifying important concepts in probabilistic biomedical text summarization. Artif Intell Med.

[2] Kirmani M, Kaur G, Mohd M (2023). ShortMail: an email summarizer system. Software Impacts.

[3] Mohd M, Wani MA, Khanday HA, Mir UB, Nasrullah S, Maqbool Z (2023). Semantic-summarizer: semantics-based text summarizer for English language text. Software Impacts.

[4] Mohd M, Jan R, Shah M (2020). Text document summarization using word embedding. Expert Syst Appl.

[5] Givchi A, Ramezani R, Baraani-Dastjerdi A (2022). Graph-based abstractive biomedical text summarization. J Biomed Inform.

[6] Lee J, Yoon W, Kim S, Kim D, Kim S, So CH, et al. BioBERT: a pre-trained biomedical language representation model for biomedical text mining. 2019; CoRR. arXiv:1901.08746. 10.1093/bioinformatics/btz682 PMC7703786 31501885

[7] Luhn HP (1958). The automatic creation of literature abstracts. IBM J Res Dev.

[8] Edmundson HP, Wyllys RE (1961). Automatic abstracting and indexing–survey and recommendations. Commun ACM.

[9] Baxendale PB (1958). Machine-made index for technical literature-an experiment. IBM J Res Dev.

[10] Afantenos S, Karkaletsis V, Stamatopoulos P (2005). Summarization from medical documents: a survey. Artif Intell Med.

[11] Bhat IK, Mohd M, Hashmy R, Pant M, Ray K, Sharma TK, Rawat S, Bandyopadhyay A (2018). SumItUp: a hybrid single-document text summarizer. Soft computing: theories and applications.

[12] Mohd M, Shah MB, Bhat SA, Kawa UB, Khanday HA, Wani AH, et al. Sumdoc: a unified approach for automatic text summarization. In: Pant M, Deep K, Bansal JC, Nagar A, Das KN, (eds). In: Proceedings of fifth international conference on soft computing for problem solving. Singapore: Springer Singapore; 2016. p. 333–343.

[13] Mihalcea R, Tarau P. Textrank: Bringing order into text. In: Proceedings of the 2004 conference on empirical methods in natural language processing; 2004.

[14] Kirmani M, Kaur G, Mohd M. Analysis of abstractive and extractive summarization methods. Int J Emerg Technol Learn. 2024;19(1).

[15] Shoaib U, Fiaz L, Chakraborty C, Rauf HT (2023). Context-aware Urdu information retrieval system. Trans Asian Low-Resource Lang. Inform. Process..

[16] Zhao L, Wu L, Huang X (2009). Using query expansion in graph-based approach for query-focused multi-document summarization. Inform Process Manag.

[17] Li W, Li W, Li B, Chen Q, Wu M. The Hong Kong polytechnic university at DUC 2005. In: Proceedings of document understanding conferences. Citeseer; 2005.

[18] Ouyang Y, Li W, Li S, Lu Q (2011). Applying regression models to query-focused multi-document summarization. Inform Process Manag.

[19] Rahman N, Borah B (2020). Improvement of query-based text summarization using word sense disambiguation. Complex Intell Syst.

[20] Sun E, Hou Y, Wang D, Zhang Y, Wang NX. D2S: Document-to-slide generation via query-based text summarization. 2021. arXiv preprint arXiv:2105.03664.

[21] Wong KF, Wu M, Li W. Extractive summarization using supervised and semi-supervised learning. In: Proceedings of the 22nd international conference on computational linguistics-Volume 1. Association for Computational Linguistics; 2008. p. 985–992.

[22] Neto JL, Freitas AA, Kaestner CA. Automatic text summarization using a machine learning approach. In: Brazilian symposium on artificial intelligence. Springer; 2002. p. 205–215.

[23] Rabiner LR, Juang BH (1986). An introduction to hidden Markov models. IEEE ASSP Magazine.

[24] Conroy JM, O’leary DP. Text summarization via hidden markov models. In: Proceedings of the 24th annual international ACM SIGIR conference on Research and development in information retrieval. ACM; 2001. p. 406–407.

[25] Dash DP, Kolekar MH, Chakraborty C, Khosravi MR (2024). Review of machine and deep learning techniques in epileptic seizure detection using physiological signals and sentiment analysis. Trans Asian Low-Res Lang Inform Process.

[26] Kågebäck M, Mogren O, Tahmasebi N, Dubhashi D. Extractive summarization using continuous vector space models. In: Proceedings of the 2nd workshop on continuous vector space models and their compositionality (CVSC); 2014. p. 31–39.

[27] Rush AM, Chopra S, Weston J. A neural attention model for abstractive sentence summarization. 2015. arXiv preprint arXiv:1509.00685.

[28] Chopra S, Auli M, Rush AM. Abstractive sentence summarization with attentive recurrent neural networks. In: Proceedings of the 2016 conference of the north american chapter of the association for computational linguistics: human language technologies; 2016. p. 93–98.

[29] Nallapati R, Zhou B, Gulcehre C, Xiang B, et al. Abstractive text summarization using sequence-to-sequence rnns and beyond. 2016. arXiv preprint arXiv:1602.06023.

[30] Gu J, Lu Z, Li H, Li VO. Incorporating copying mechanism in sequence-to-sequence learning. 2016. arXiv preprint arXiv:1603.06393.

[31] See A, Liu PJ, Manning CD. Get to the point: summarization with pointer-generator networks. 2017. arXiv preprint arXiv:1704.04368.

[32] Hu B, Chen Q, Zhu F. Lcsts: A large scale chinese short text summarization dataset. 2015. arXiv preprint arXiv:1506.05865.

[33] Chen Q, Zhu X, Ling Z, Wei S, Jiang H. Distraction-based neural networks for document summarization. 2016. arXiv preprint arXiv:1610.08462.

[34] Ma S, Sun X, Li W, Li S, Li W, Ren X. Query and output: Generating words by querying distributed word representations for paraphrase generation. In: Proceedings of the 2018 Conference of the North American Chapter of the Association for Computational Linguistics: Human Language Technologies, Volume 1 (Long Papers). vol. 1; 2018. p. 196–206.

[35] Paulus R, Xiong C, Socher R. A deep reinforced model for abstractive summarization. 2017. arXiv preprint arXiv:1705.04304.

[36] Mao X, Yang H, Huang S, Liu Y, Li R (2019). Extractive summarization using supervised and unsupervised learning. Expert Syst Appl.

[37] Amancio DR, Silva FN, da F Costa L (2015). Concentric network symmetry grasps authors’ styles in word adjacency networks. EPL (Europhys Lett)..

[38] Tohalino JV, Amancio DR (2018). Extractive multi-document summarization using multilayer networks. Physica A.

[39] Chakraborty C, Dash TK, Panda G, Solanki SS (2022). Phase-based cepstral features for automatic speech emotion recognition of low resource Indian languages. Trans Asian Low-Res Lang Inform Process.

[40] Moradi M, Ghadiri N. Text summarization in the biomedical domain. 2019. arXiv preprint arXiv:1908.02285.

[41] Gigioli P, Sagar N, Rao A, Voyles J. Domain-aware abstractive text summarization for medical documents. In: 2018 IEEE International conference on bioinformatics and biomedicine (BIBM). IEEE; 2018. p. 2338–2343.

[42] Kieuvongngam V, Tan B, Niu Y. Automatic text summarization of covid-19 medical research articles using bert and gpt-2. 2020. arXiv preprint arXiv:2006.01997.

[43] Raffel C, Shazeer N, Roberts A, Lee K, Narang S, Matena M (2020). Exploring the limits of transfer learning with a unified text-to-text transformer. J Mach Learn Res.

[44] Manning CD, Surdeanu M, Bauer J, Finkel J, Bethard SJ, McClosky D. The Stanford CoreNLP Natural Language Processing Toolkit. In: Association for computational linguistics (ACL) System Demonstrations; 2014. p. 55–60. Available from: http://www.aclweb.org/anthology/P/P14/P14-5010.

[45] Lee J, Yoon W, Kim S, Kim D, Kim S, So CH (2020). BioBERT: a pre-trained biomedical language representation model for biomedical text mining. Bioinformatics.

[46] Mohd M, Hashmy R. Question classification using a knowledge-based semantic kernel. In: Soft computing: theories and applications: proceedings of SoCTA 2016, Volume 1. Springer; 2018. p. 599–606.

[47] Kim D, Lee J, So CH, Jeon H, Jeong M, Choi Y (2019). A neural named entity recognition and multi-type normalization tool for biomedical text mining. IEEE Access.

[48] Lin C, Miller T, Dligach D, Bethard S, Savova G. A BERT-based universal model for both within-and cross-sentence clinical temporal relation extraction. In: Proceedings of the 2nd clinical natural language processing workshop; 2019. p. 65–71.

[49] Mohd M, Javeed S, Wani MA, Khanday HA, Wani AH, Mir UB (2023). poliWeet-Election prediction tool using tweets. Software Impacts.

[50] Moen S, Ananiadou TSS. Distributional semantics resources for biomedical text processing. Proceedings of LBM. 2013;p. 39–44.

[51] Mikolov T, Chen K, Corrado G, Dean J. Efficient estimation of word representations in vector space. arXiv preprint arXiv:1301.3781. 2013.

[52] Devlin J, Chang MW, Lee K, Toutanova K. Bert: pre-training of deep bidirectional transformers for language understanding. 2018. arXiv preprint arXiv:1810.04805.

[53] Mohd M, Javeed S, Nowsheena Wani MA, Khanday HA (2022). Sentiment analysis using lexico-semantic features. J Inform Sci.

[54] Hartigan JA, Wong MA (1979). Algorithm AS 136: a k-means clustering algorithm. J Roy Stat Soc: Ser C (Appl Stat).

[55] Mishra R, Bian J, Fiszman M, Weir CR, Jonnalagadda S, Mostafa J (2014). Text summarization in the biomedical domain: a systematic review of recent research. J Biomed Inform.

[56] Plaza L, Díaz A, Gervás P (2011). A semantic graph-based approach to biomedical summarisation. Artif Intell Med.

[57] Barrios F, López F, Argerich L, Wachenchauzer R. Variations of the similarity function of textrank for automated summarization. arXiv preprint arXiv:1602.03606. 2016.

[58] Zhang J, Wang T, Wan X. PKUSUMSUM: a Java platform for multilingual document summarization. In: Proceedings of COLING 2016, the 26th International conference on computational linguistics: system demonstrations; 2016. p. 287–291.

[59] Ganesan K. ROUGE 2.0: Updated and improved measures for evaluation of summarization tasks. arXiv preprint arXiv:1803.01937. 2018.

[60] Dash TK, Chakraborty C, Mahapatra S, Panda G (2022). Mitigating information interruptions by COVID-19 face masks: a three-stage speech enhancement scheme. IEEE Trans Comput Social Syst.

[61] Dash TK, Chakraborty C, Mahapatra S, Panda G (2022). Gradient boosting machine and efficient combination of features for speech-based detection of COVID-19. IEEE J Biomed Health Inform.

